# Impact of chain-length of sulfhydryl-modified surface-decorated surfactants on mucoadhesive nanostructured lipid carriers

**DOI:** 10.1007/s13346-025-01905-w

**Published:** 2025-06-24

**Authors:** Samia Kausar, Sofia O. D. Duarte, Ahmed Raza Hashmi, Farwa Zahra, Alia Erum, Shumaila Arshad, Ume Ruqia Tulain, Mulazim Hussain Asim, Pedro Fonte

**Affiliations:** 1 College of Pharmacy, University of Sargodha, Sargodha, Punjab 40100 Pakistan; 2 Lahore College of Pharmaceutical Sciences, Raiwind road, Lahore, 54000 Pakistan; 3 iBB– Institute for Bioengineering and Biosciences, Department of Bioengineering, Instituto Superior Técnico, Universidade de Lisboa, Av. Rovisco Pais 1, Lisboa, 1049-001 Portugal; 4 Associate Laboratory i4HB—Institute for Health and Bioeconomy, Instituto Superior Técnico, Universidade de Lisboa, Av. Rovisco Pais, Lisboa, 1049-001 Portugal; 5 School of Pharmacy, Monash University Malaysia, Bandar Sunway, Subang Jaya, Selangor Malaysia; 6 Doctorʹs Institute of Health Sciences, Sargodha, 40100 Pakistan; 7 ILM College of Pharmaceutical Sciences & Sargodha College of Medical Sciences, Sargodha, 40100 Pakistan; 8 Department of Chemistry and Pharmacy, Faculty of Sciences and Technology, University of Algarve, Gambelas Campus, Faro, 8005-139 Portugal; 9 Centro de Ciências do Mar do Algarve (CCMAR/CIMAR LA), Universidade do Algarve, Campus de Gambelas, Faro, 8005-139 Portugal

**Keywords:** Mucoadhesive NLCs, Mucosal permeability, Sulfhydryl-modified surfactants, Aprepitant, Controlled drug release, Enhanced oral bioavailability

## Abstract

Nanostructured lipid carriers (NLCs) decorated with sulfhydryl-modified surfactants have recently gained attention for delivering BCS Class IV drugs. However, the impact of the chain-length of these surfactants on the permeation and bioavailability properties of NLCs is still unknown. Therefore, this study investigates the effect of surfactant chain-length on the mucoadhesive, permeation, and bioavailability properties of NLCs. For this purpose, short- and long-chain sulfhydryl-modified polyethoxylated surfactants were generated to develop mucoadhesive NLCs and loaded with the model drug aprepitant (APT). NLCs were characterized and assessed for comprehensive physicochemical and biological evaluations. Moreover, in-vivo studies were performed for proof-of-concept to show enhanced oral drug bioavailability. NLCs showed particle size under 200 nm with 6.9 and 6.7% drug loading and 85 and 84% drug entrapment for short- and long-chain surfactants, respectively. The drug-loaded NLCs were safe and stable, and short- and long-chain surfactants containing NLCs exhibited 11.6- and 9.6-fold enhanced mucoadhesion, respectively. Moreover, in comparison to long-chain sulfhydryl-modified surfactant, short-chain surfactant is transported into deeper segments of mucus due to less interaction with the mucus. Similarly, short-chain sulfhydryl-modified surfactants showed significantly enhanced cellular permeation across Caco-2 cell lines. Furthermore, the long-chain sulfhydryl-modified surfactants showed 4.38-fold enhanced C_max_, whereas due to better diffusion and mucoadhesion properties, the short-chain surfactants exhibited 5.38-fold enhanced C_max_. Similarly, 34.8% relative bioavailability was attained for short-chain surfactants and 24.8% for long-chain surfactants. These results suggest short-chain sulfhydryl surfactants are promising candidates for improving the oral delivery of poorly soluble drugs and warrant further investigation for clinical translation.

**Graphical Abstract:**

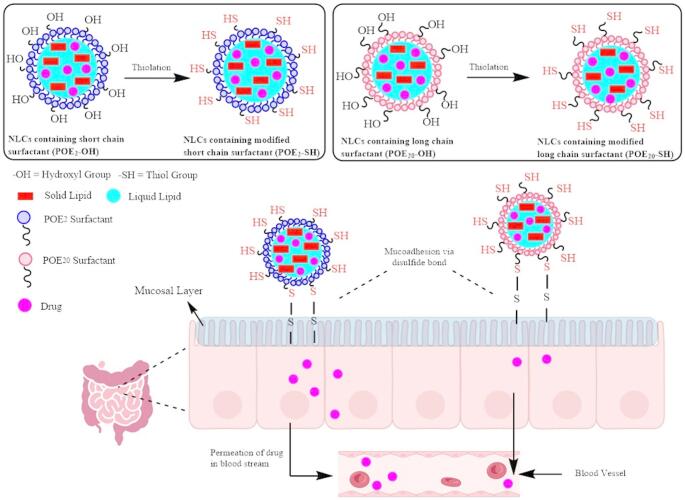

**Supplementary Information:**

The online version contains supplementary material available at 10.1007/s13346-025-01905-w.

## Introduction

Different lipid-based nanocarriers are nowadays being used in drug delivery systems, in which drugs are entrapped for improving drug delivery of BCS Class IV drugs [1, 2]. Lipid-based nanoparticles like liposomes, nano-emulsions, micro-emulsions, polymeric nanoparticles [3], polymeric micelles [4], solid lipid nanoparticles (SLNs), self-nanoemulsifying systems, self-microemulsifying systems and nanostructured lipid carriers (NLCs) have been commonly used [5]. However, SLNs and NLCs are considered more promising lipid carriers to overcome the drawbacks of oral drug delivery. However, a few limitations of SLNs make NLCs an ideal carrier for oral drug delivery [6]. NLCs are second-generation lipid nanoparticles that are designed via a combination of both solid and liquid lipids [7]. The combination of solid and liquid lipids creates a matrix that improves drug loading and stability of NLCs [8]. However, NLCs also have the shortcoming of poor GIT residence time due to its non-mucoadhesive nature.

In our previous study, we developed mucoadhesive NLCs using long-chain sulfhydryl polyoxyethylene 20-oleyl ether surfactant (POE_20_-SH) and evaluated its impact on enhancing mucoadhesion, permeation and oral bioavailability [9]. However, the impact of sulfhydryl-modified surfactants chain-length on mucoadhesive, permeation and bioavailability has not been previously explored. Therefore, in the current study, we compare the impact of the chain-length of different sulfhydryl-modified polyethoxylated surfactants on the permeation and bioavailability of poorly soluble drugs in mucoadhesive NLCs. For this purpose, we generated a short-chain sulfhydryl-modified polyoxyethylene 2-oleyl ether surfactant (POE_2_–SH) for the development of mucoadhesive NLCs and compared this with the mucoadhesive NLCs developed by using longer-chain sulfhydryl-modified polyoxyethylene 20-oleyl ether surfactant (POE_20_-SH). Moreover, designed formulations were loaded with aprepitant, a selective antagonist for neurokinin-1 (NK-1) receptor drug [10, 11]. Aprepitant (BCS Class IV drug) shows poor solubility and permeability, leading to reduce drug bioavailability [12]. Different formulations were characterized, and in-vitro and in-vivo studies were performed to confirm the impact of different chain-lengths of surfactants on the different properties of drug-loaded mucoadhesive NLCs.

## Materials and methods

### Materials

N-Bromosuccinimide (NBS), triphenylphosphine (Ph_3_P), lithium bromide, Span 60 (melting point 53 °C), thiourea, stearic acid (melting point 69–71 °C), polyethylene glycol 6000 (PEG 6000), dichloromethane (CH_2_Cl_2_), and Ellman’s reagent were purchased from Merck, USA. Polyoxyethylene (2) oleyl ether (Brij 93) and polyoxyethylene (20) oleyl ether (Brij 98) were obtained from Shanghai Macklin Biochemical Co., Ltd. China and miglyol was collected from Merck, Austria. Labrasol was kindly donated by Gattefossé, France. Dimethylformamide (DMF) and diethyl ether were purchased from Riedel-de-Haen, Germany. Aprepitant was generously donated by Ferozsons Laboratories Limited, Lahore, Pakistan. The PVDF filters (0.22 μm) were acquired from Starlab, UK. Dialysis membrane spectra (molecular weight cut-off 3.5 kDa) were purchased from Carl Roth GmbH, Karlsruhe, Germany.

Minimum essential medium powder was synthesized using fetal bovine serum (10%), Earle’s salts, 2 mM L-glutamine, sodium bicarbonate, phenol red and penicillin-streptomycin solution. All other chemicals used in this study were of analytical grade.

### Synthesis of sulfhydryl-modified POE_2_/POE_20_ (POE_2_-SH/POE_20_-SH)

After slight modification in our pre-established procedure to optimize reaction conditions, sulfhydryl modification of POE_2_-OH and POE_20_-OH was carried out to attain POE_2_-SH and POE_20_-SH, respectively [13]. In detail, Ph_3_P and NBS were dried in a desiccator under low pressure and passed through a 4 Å sieve. In the first step of bromination, 5 mL of CH_2_Cl_2_ was used to dissolve 1.0 mM of Ph_3_P and 1.1 mM of NBS and in the following 2 mM LiBr and POE_2_-OH/POE_20_-OH were added to the resulting solution, and the solution was kept stirring overnight at 40 ºC. Afterwards, the solution was cooled to room temperature and an equivalent concentration of diethyl ether was added to attain triphenylphosphine oxide precipitates. These precipitates were removed via centrifugation at 4 ºC for 15 min at 13,000 rpm. In the following, the resulting solution was vacuum dried via rotavapor, and the brominated product (POE_2_-Br/POE_20_-Br) was obtained as clear oil.

In next step, halogens were substituted by–SH groups via substitution reaction in the presence of thiourea. In detail, halogenated intermediate products were dissolved in DMF and 5 mg/mL of thiourea dissolved in DMF was added to it. DMF was separated from the resulting solution after overnight stirring at 90 ºC. The resultant product was purified via column chromatography using a stationary phase of silica gel 60 (230–400 mesh) and acetone and dichloromethane in an equal proportion as a mobile phase. After that, appropriate fractions were removed via rotavapor; yielding sulfhydryl-modified surfactants (POE_2_-SH and POE_20_-SH) as a waxy material and confirmed for further characterization [13].

### Characterization of sulfhydryl-modified surfactants with FTIR, ^1^H NMR and Ellman’s essay

The FTIR (IRPrestige-21) equipment was used for the characterization analysis of the POE_2_-SH and POE_20_-SH. Data was established in the domain of 4000 cm^− 1^ to 400 cm^− 1^ as the expected peaks associated with sulfhydryl groups (e.g., S-H stretch around 2550–2600 cm⁻¹) fall in this range. Moreover, ^1^HNMR spectra were obtained via Varian Gemini 200 spectrometer using CDCl_3_ as solvent. Chemical shifts were referenced by using the solvent residual peak of CDCl_3_. Furthermore, Ellman’s test was conducted to verify the sulfhydryl modification of POE_2_-OH/POE_20_-OH [13, 14]. In detail, 1 mg of POE_2_-SH/POE_20_-SH solution was dissolved to 0.5 mL of 1 M PBS at pH 7.4 and added to the freshly prepared 0.5 mL Ellman’s reagent. At 25 °C, resultant solution was incubated in a light-protected chamber for 2 h. Subsequently, at 13,000 rpm the solution was centrifuged for 5 min. Ellman’s reagent reacts with sulfhydryl groups to form a yellow-colored product, enabling quantitative measurement that was monitored at 450 nm absorbance.

### Synthesis of mucoadhesive drug-loaded NLCs

The nano-template engineering method was applied to design NLCs with slight modification in temperature and stirring speed in the pre-established protocol [15]. As shown in Table 1, a blend of Span 60, labrasol, miglyol, PEG 6000 and stearic acid were melted at 80 ºC using a thermomixer at 500 rpm and 10 mL of pre-heated water was added to the resultant mixture. After uninterrupted stirring for at 500 rpm for 45 min at 80 ºC, a nano-emulsion was generated. In the following, aprepitant (3 mg) and 5 mL of sulfhydryl-modified surfactant (POE_2_-SH/POE_20_-SH) solutions were added to the nano-emulsion and incubated for 5 min at 80 ºC. The mucoadhesive drug-loaded NLCs were generated by rapid cooling in ice water. Finally, the formulation was filtered using a 0.22 μm micro-filter and stored at 4 ºC for further characterization [16]. Additionally, drug-loaded NLCs using un-modified surfactant were also synthesized for comparison with mucoadhesive drug-loaded NLCs.

**Table 1:** Formulation design of different mucoadhesive drug-loaded NLCs

Formulations	Labrasol (mg)	Miglyol (mg)	Span 60 (mg)	Stearic acid (mg)	PEG 6000 (mg)	POE_2_-SH/ POE_20_-SH (mg)	Aprepitant (mg)
NLCs-I	6.0	2.0	0.5	2.0	6.0	10	2.0
NLCs-II	6.0	1.0	1.0	2.0	6.0	10	2.0
NLCs-III	6.0	2.0	1.5	1.0	6.0	10	2.0
NLCs-IV	4.0	1.0	1.0	2.0	5.0	10	2.0

### HPLC quantification of APT

Quantification of aprepitant was done via HPLC following a pre-established procedure [17] using Hitachi LaChrom Elite HPLC-System that was equipped with a diode array detector (L-2450), autosampler (L-2200), and a pump (L-2130). In short, a blend of acetonitrile and orthophosphoric acid (0.1%) in 60:40 was used as a mobile phase and Ascentis column (15 cm × 4.6 mm, 5 μm) was employed as a stationary phase. During the process, the flow rate of 1.0 mL/min and an injection volume of 10 µL were maintained. For the calibration curve, standard APT solutions (0.2–100 µg/mL) were used and the UV detector was set on a wavelength of 210 nm [18].

### Characterization of NLCs

#### Zeta potential (ζ), particle size and polydispersity index (PDI)

The mean diameter, size distribution, and ζ potential values of mucoadhesive drug-loaded NLCs were assessed using Anton Paar Particle Size Analyzers, (Austria). Samples were diluted with water and analyzed at 25 °C. Dimensions and size distribution were assessed using dynamic light scattering. The size distribution was characterized by the polydispersity index (PDI), and the average particle size was expressed as the intensity mean diameter. The ζ potential measurements were conducted following the dilution of the sample in a freshly prepared aqueous KCl solution (1 mM).

### Entrapment efficiency and drug loading capacity

The entrapment efficiency (EE) assures entrapment of drug in the nanocarriers, whereas the drug loading capacity (LC) is determined by dividing the quantity of drug entrapped by the total quantity of drug used [19].

In the liquid phase, the free amount of drug was monitored to evaluate the entrapment and drug loading of mucoadhesive APT-loaded NLCs. After dilution, the suspension (5 mL) was heated for 5 min at 45 ºC. Subsequently, the suspension was centrifuged, filtered using PVDF filters (pore size 0.22 μm) and drug concentration was measured via HPLC analysis. The entrapment and drug loading were calculated via Eqs. 1 and 2, respectively.

1 $$\eqalign{& \% {\rm{ Entrapment \ efficiency }}(\% {\rm{EE}}) = \cr & {{{\rm{ Total \ drug \ in \ NLCs }} - {\rm{ Free \ drug }}} \over {{\rm{ Total \ drug }}}} \times 100 \cr} $$

2 $$\eqalign{& \% {\rm{ Loading \ capacity }}(\% {\rm{LC}}) = \cr & {{{\rm{ Quantity \ of \ drug \ in \ NLCs }}} \over {{\rm{ Total \ weight \ of \ drug }}}} \times 100 \cr} $$

### In-vitro drug release studies

The dialysis membrane technique was used to assess the in-vitro drug release profile [19, 20]. In detail, for activation of dialysis bag (MWCO 3.5 kDa), it was submerged for 24 h in a release medium (PBS). Subsequently, 5 mL of free drug, APT-loaded NLCs and mucoadhesive APT-loaded NLCs (POE_2_-SH/POE_20_-SH) were filtered (PVDF filter of 0.22 μm pore size). Afterwards, solutions were shifted into the dialysis bag and sealed with clips. The bag was allowed to settle in the beaker containing 250 mL release medium at 37 ºC and the medium was continuously stirred at 100 rpm. Moreover, to maintain sink conditions Tween 80 (2% w/v) was added. The 1 mL samples were taken at intervals of 4 h for 24 h, and an equal volume of fresh medium was added. To calculate the drug released, samples were analyzed via HPLC method.

### Stability studies

To evaluate the storage stability of the formulations, mucoadhesive APT-loaded NLCs decorated with POE_2_-SH and POE_20_-SH were kept at accelerated stability conditions (4 ºC and 25 ºC) for 1, 3, 4 and 6 months under controlled humidity conditions. Afterward, to examine the stability of formulations, entrapment efficiency and particle size were determined [21]. Moreover, containing short- and long chain thiolated surfactants was assessed in simulated gastric fluid (pH 1.2) and simulated intestinal fluid (pH 6.8) that were prepared in accordance with the USP guidelines. For this purpose, 100 mg NLCs were dispersed in 100 mL of simulated fluids with continuous stirring at 37 °C. After 1, 2, 3, 4, and 6 h, samples were collected, and size and zeta potential were measured [22].

### Safety studies

To monitor the safety of mucoadhesive APT-loaded NLCs (POE_2_-SH/POE_20_-SH), resazurin assay was performed via Caco-2 cell lines [23–25]. The cells were cultivated in MEM on a 24-well plate at 37 ºC and the medium was re-freshed daily. After 80% cells confluency, they were used for experimental analysis. First, cells were rinsed with warmed PBS and then the cell culture was enriched with 500 µL of APT suspension, 0.5% (v/v) APT-loaded NLCs, mucoadhesive APT-loaded NLCs containing POE_2_-SH or POE_20_-SH surfactants, a negative test control 1% (v/v) Triton X-100 and positive test control (MEM without phenol red). After 3 and 24 h, the sample solutions were removed, and PBS was used to rinse the cells. After this, the medium was changed with 0.5 mL of the resazurin solution and incubated for 3 h. A 96-well plate was loaded with 100 µL sample and at 540 nm fluorescence intensity was monitored. To determine cell viability, the following equation was employed:

$$\eqalign{& {\rm{ Cell}}\;{\rm{Viability }}(\% ) = \cr & {{{\rm{ Experimental}}\;{\rm{value }} - {\rm{ Negative}}\;{\rm{test}}\;{\rm{control }}} \over {{\rm{ Positive}}\;{\rm{test}}\;{\rm{control }} - {\rm{ Negative}}\;{\rm{test}}\;{\rm{control }}}} \times 100 \cr} $$

### Mucoadhesion evaluation

An already developed half-cut falcon tube technique was used to examine the mucoadhesive behavior of newly synthesized short- and long-chain sulfhydryl-modified surfactants containing mucoadhesive NLCs [26].

In detail, a fresh intestinal mucosa of goat was collected from the local slaughterhouse and cut longitudinally. Afterwards, mucosa was treated with phosphate buffer (pH 7.4) and divided into smaller fragments (5 × 2 cm). The mucosae were fixed on half-cut Falcon tubes at 45º in the incubation chamber at 37 ºC with 100% relative humidity. The drug-loaded NLCs and mucoadhesive drug-loaded NLCs decorated with POE_2_-SH and POE_20_-SH containing 3 mg loaded drug were uniformly applied at the mucosal surfaces. Moreover, 3 mg of free drug was applied on intestinal mucosal surface that served as a control. Mucosal surface was flushed for 3 h with phosphate buffer saline solution and at specific time the wash-off was collected and the drug concentration was estimated via HPLC analysis.

### Rheological studies

Viscosity evaluation of newly synthesized mucoadhesive NLCs containing short- and long-chain sulfhydryl-modified surfactants was performed to explore the interaction of mucus with these NLCs formulations. For this purpose, mucus was gathered from the intestine surface of goat mucosa. For purification of mucus, it was diluted with NaCl (0.1 M) in 1ː5 (w/v) and stirred slowly at 0 °C for 1 h, centrifuged at 10,400 x g for 2 h and supernatant was removed. The process was repeated with half volume of NaCl used before and purified mucus was obtained.

The purified mucus (500 µL) was mixed with different mucoadhesive NLCs formulations in a 1:2 with the help of spatula and incubated for 1 and 4 h at 37 °C. In the following, samples were shifted to a Haake Mars plate-plate rheometer (Thermo Scientific, Austria) to calculate viscosity. Purified blank mucus mixed with PBS served as control [27]. The samples were prepared in triplicate to ensure consistency.

### Permeation studies

#### Rotating tube method

Mucoadhesive NLCs (POE_2_-SH and POE_20_-SH) were examined via rotating tube technique in order to monitor the diffusion/permeation properties [9]. In detail, silicon tubes were divided into small fragments (3 cm length) having an internal diameter of 30 mm. After sealing one end of the tube, 100 µL of mucus was loaded via syringe through the other end. The 50 µL of 0.1% (v/v) unmodified surfactant containing NLCs and mucoadhesive NLCs (POE_2_-SH and POE_20_-SH) were inserted into mucus-loaded silicon tubes through open end. Moreover, NLCs were florescence-labeled with LFR fluorescent (0.05% w/v) that is soluble in DMF and can be measured at 570 nm and 610 nm excitation and emission wavelength, respectively. Afterward, tubes were spun for 24 h at 37 ºC at 50 rpm under dark and frozen at -80 ºC. Subsequently, tubes were cut into 3 mm equal 10 segments, treated with 10 mM PBS (pH 6.8) solution for half an hour and centrifuged at 13,000 rpm for 5 min. In the end, 100 µL of each sample was collected and its wavelength was monitored. The results were calculated as a fluorescence percentage in all the segments of the tube.

#### Cellular permeation studies

To further confirm permeation ability of short- and long-chain sulfhydryl-modified surfactants containing NLCs, cellular diffusion studies were performed using Caco-2 cell lines using 24-transwell plate having 1.13 cm^2^ permeation area [28]. Caco-2 cells are a standard model for intestinal permeability, and TEER values ensure the integrity of the monolayer. Experiment was conducted when cell confluency of 2.5 × 10^4^ cells per well was achieved. Media was re-freshed on alternative days using minimum essential medium (MEM) without phenol red. Before start of the study, TEER (transepithelial electrical resistance) value of cell monolayer was calculated and cell wells with TEER value ˃800 Ωcm^2^ were chosen for the study. A model dye Lucifer yellow in 0.1% (m/v) concentration was added to the donor chambers along with100 µL of 0.05% (m/v) formulations. Moreover, Lucifer yellow in MEM solution was used as a control. The samples were put in an incubator at 37 °C for 3 h and fluorescence intensity was measured after each half hour at a λ_ex_ of 434 nm and λ_em_ of 540 nm. Results were calculated as percentage Lucifer yellow diffused across cells. The apparent permeability coefficient (P_app_) for Lucifer yellow was measured using the following equation:

$${{\rm{P}}_{{\rm{app}}}} = {\rm{Q}}/\;{\rm{A}}\; \times \;{\rm{c}}\; \times \;{\rm{t}}$$

Where “Q” stands for the total quantity of dye diffused within 3 h, “A” is the permeation area, “c” is the quantity of the dye used, and “t” is the duration of the study. The permeation enhancement ratio (R) was calculated as given below:

$$\:\mathrm{R=}\frac{{\mathrm{P}}_{\mathrm{app}}\mathrm{(Sample)}}{{\mathrm{P}}_{\mathrm{app}}{\:(Control)}}$$

### In-vivo studies

#### Animal’s selection and dosing

For in-vivo studies, all necessary protocols were followed in line with the principles of the Declaration of Helsinki, which were approved by the Institutional Animal Ethical Committee, College of Pharmacy, University of Sargodha, Pakistan (Approval No. SU/ORIC/2098). For this purpose, 5 groups of Sprague-Dawley rats (5 rats in each group) were selected with an average weight of 300–350 g. The first group was selected for APT suspension and the second, third and fourth groups were chosen for APT-loaded NLCs, mucoadhesive APT-loaded NLCs (POE_2_-SH) and mucoadhesive APT-loaded NLCs (POE_20_-SH), respectively. Formulations were administered orally via gavage in groups 1–4. The fifth group received an intravenous dose of APT in sterile phosphate buffer saline solution at pH 7.4. The sufficient amount of water was available before dosing, but no diet was given 2 h before dosing. A dose of 2 mg/kg body weight was delivered to each group rats in1.5–2 mL dosing volume [29]. The blood samples of 0.2 mL were obtained in EDTA tubes at 1, 2, 3, 5, 8 and 24 h.

#### Pharmacokinetic evaluation

Blood samples were centrifuged for 20 min at 3000 rpm and the plasma top layer was removed. In the following, 0.9 mL of acetonitrile was added to 1 mL of plasma to precipitate proteins, and the resulting solution was centrifuged again for 15 min at 3500 rpm. The supernatant layer was removed, evaporated, and evaluated via HPLC. PKSolver software was employed to compute the AUC, C_max_, and T_max_. The relative bioavailability of mucoadhesive APT-loaded NLCs (POE_2_-SH and POE_20_-SH) was calculated as follows:

$$\eqalign{& \% {\rm{ Relative \ bioavailability }}\left( {\% \;{{\rm{F}}_{\rm{r}}}} \right) \cr & = {{{{[{\rm{AUC}}]}_{{\rm{oral }}}}} \over {{{[{\rm{AUC}}]}_{{\rm{iv }}}}}} \times {{{{[{\rm{D}}]}_{{\rm{iv }}}}} \over {{{[{\rm{D}}]}_{{\rm{oral }}}}}} \times 100 \cr} $$

### Statistical analysis

For statistical investigation, a student’s t-test was performed. A confidence interval of *p* < 0.05 was chosen for the analysis. The study engaged a one-way ANOVA with post hoc Tukey’s multiple-comparisons test, having a p value < 0.05 being significant (GraphPad Prism, GraphPad Software, Inc.). The outcomes were presented as the mean (± SD), *n* = 3.

## Results and discussion

### Synthesis of POE_2_-SH/POE_20_-SH

**Fig. 1:**
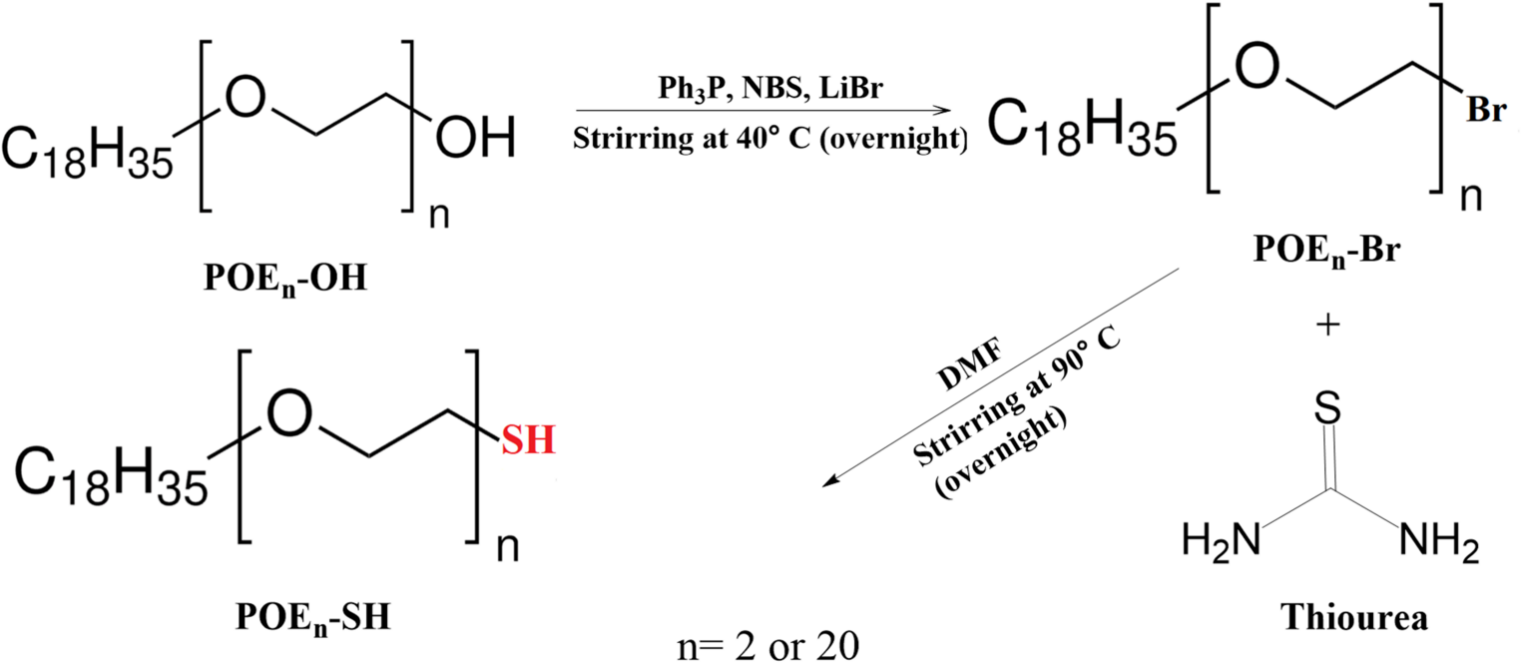
Sulfhydryl modification of POE_2_/POE_20_: Initially, bromination of POE_2_/POE_20_ was performed with LiBr using a brominating agent, NBS and a dehydrating agent, Ph_3_P dissolved in DMF that produced a brominated intermediate product and in the next step, thiourea was used to substitute bromine with sulfhydryl groups to generate POE_2_-SH/POE_20_-SH

The unmodified surfactants are not mucoadhesive at all, so surfactants were modified to generate mucoadhesive surfactants to achieve the desire characteristics required for this research work. Therefore, for the development of POE_2_-SH and POE_20_-SH, terminal hydroxyl groups of surfactants were first substituted with bromine moieties, and then thiourea was employed to replace–Br with thiol groups via a nucleophilic substitution reaction as demonstrated in Fig. 1.

Most of the molecules are highly susceptible to nucleophilic reactions, therefore, bromination of POE_2_-SH was done using NBS (a brominating agent) [14]. Moreover, the reaction was conducted at low temperature to prevent the deterioration of the product. The amount of reagents and type of solvent used during the chemical reaction was regulated to maintain the reaction conditions. Specifically, the concentration of DMF is substantial because it has an influence on the bromination process. Hence, an appropriate solvent concentration was found during trials. In the end, an isothiuronium salt was constituted by the reaction of thiourea with halides that was hydrolysis to yield a waxy, white sulfhydryl-modified surfactant with 90% yield [14, 30].

### Characterization through FT-IR, ^1^H NMR and Ellman’s essay

FT-IR examination was performed to verify the existence of the–SH groups on the surface of POE_2_-SH and POE_20_-SH as illustrated in Supplementary Figures S1 and S2, respectively. The terminal hydroxyl groups of unmodified surfactants (POE_2_-OH and POE_20_-OH) depicted an extensive characteristic peak around 3450 cm^− 1^ and 3161 cm^− 1^, respectively. A robust peak around 2339.65 cm^− 1^ and 2889 cm^− 1^ in POE_2_-OH and POE_20_-OH, respectively associated with CH- stretching. The C-O-C and C-OH stretching are accountable for prominent peaks at 933.5 cm^− 1^ of POE_2_-OH and at 1099 cm^− 1^ of POE_20_-OH. Moreover, additional peaks at around 682.80 cm^− 1^ for POE_2_-SH and at 561 cm^− 1^ for POE_20_-SH are correlated with the C-S group vibrational stretching. The existence of these peaks revealed sulfhydryl modification [9]. Moreover, ^1^HNMR spectra confirmed the structure of short- and long-chain thiolated surfactants as expressed in Supplementary Figures S3 and S4. In the spectrum of POE_2_-SH and POE_20_-SH, the methylene protons are present at 3.50 ppm, whereas peak at 1.4 ppm confirmed the signal of the sulfhydryl groups.

Additionally, sulfhydryl modification of the surfactants was confirmed via Ellman’s assay and results confirmed 1897 and 819 µmol/g–SH groups at POE_2_-SH and POE_20_-SH, respectively.

### Preparation of mucoadhesive APT-loaded NLCs

NLCs are considered as 2nd generation lipid carriers as they contain both liquid and solid lipids [31]. However, the prospective use of NLCs is not yet fully achieved due to the lack of mucoadhesive behavior [32]. The sulfhydryl-modified surfactants were incorporated into the NLCs to improve their mucoadhesive features and to prompt the gastric residence time via disulfide bond formation between mucus glycoprotein and sulfhydryl-modified surfactant. This will lead to enhanced bioavailability of poorly soluble drugs through improved mucoadhesion and prolonged gastric residence.

The nano-template engineering technique was used to produce NLCs in which miglyol and stearic acid were utilized as liquid and solid lipids, respectively [33], whereas Span 60 was used as lipophilic, and labrasol was used as a hydrophilic surfactant [34]. Moreover, to reduce the interfacial tension between particles, PEG-6000 was utilized as a stabilizer [35]. Similarly, mucoadhesive NLCs were produced with the addition of the drug.

### Characterization of mucoadhesive APT-loaded NLCs

#### Zeta potential (ζ), particle size and polydispersity index (PDI)

These parameters were evaluated to ensure the stability, uniformity, and permeability of the NLCs, which are critical for their mucoadhesive and drug delivery properties. Tables 2, 3 displays the ζ, particle size and PDI of surface-decorated APT-loaded NLCs containing short- and long-chain sulfhydryl-modified surfactants. The size of particles play a vital role in mucus permeability and the stability of colloidal dispersion is influenced by zeta potential [36]. Four distinct types of formulations were designed by incorporating different ingredients at varying concentrations. The NLCs-II formulations containing sulfhydryl-modified short- and long-chain surfactants were selected for further studies due to the appropriate size (˂ 200 nm), and zeta potential values (-23 and − 28 mV for POE_20_-SH and POE_2_-SH). The ζ, size, and PDI values influence the mucoadhesive properties, stability, and potential for mucus permeability. For instance, more negative zeta potential values indicate improved colloidal stability, smaller particle size enhances mucus penetration and lower PDI reflects better uniformity of the formulation.

The mucoadhesive APT-loaded NLCs (NLCs-II) exhibited the zeta potential of -23.5 ± 3.2 mV and − 28.5 ± 1.3 mV for POE_20_-SH and POE_2_-SH, respectively. The high zeta potential value facilitates electrostatic repulsion between similar charges and prevents clumping of particles that provide stability to the colloidal dispersion system [34, 37]. The acquired value of PDI was 0.190 for POE_20_-SH and 0.161 for POE_2_-SH decorated NLCs, which reflects the uniform distribution of nanoparticles [38].

**Table 2:** Zeta potential (ζ), particle size and polydispersity index of mucoadhesive APT-loaded NLCs decorated with POE_2_-SH and POE_20_-SH

Formulations	ζ (mV)		Size (nm)		PDI	
	POE_20_-SH	POE_2_-SH	POE_20_-SH	POE_2_- SH	POE_20_- SH	POE_2_- SH
NLCs-I	-6.93 ± 2.13	-8.13 ± 2.19	201 ± 18	207 ± 11	0.40 ± 0.02	0.31 ± 0.01
NLCs-II	-23.5 ± 3.21	-28.5 ± 1.45	185 ± 12	144 ± 14	0.19 ± 0.01	0.16 ± 0.01
NLCs-III	-6.60 ± 5.14	-7.60 ± 2.34	221 ± 20	214 ± 17	0.94 ± 0.03	0.89 ± 0.03
NLCs-IV	-5.85 ± 4.27	-7.41 ± 1.28	233 ± 17	242 ± 10	1.29 ± 0.02	1.17 ± 0.02

### Entrapment efficiency and drug loading capacity

The highest entrapment efficiency of the drug within the lipid matrix of NLCs depends upon the optimal ratio of solid lipid to liquid lipid mixture. NLC-III demonstrated the highest drug entrapment efficiency; hence, it was chosen for the in-vitro and in-vivo investigation along with size and zeta potential values [39]. The principal interactions facilitating the encapsulation of drugs within NLCs are hydrophobic interactions and electrostatic interactions. Moreover, the driving force is to minimize lipid surface area and solubilize the drug within the lipid core [40].

To ensure efficient drug incorporation and optimal dosage delivery, freshly prepared mucoadhesive APT-loaded NLCs decorated with POE_2_-SH and POE_20_-SH in aqueous dispersions were investigated for drug loading and entrapment. The drug contents were evaluated via HPLC analysis and results showed 85% and 86% drug entrapment for POE_2_-SH and POE_20_-SH decorated NLCs, respectively. Moreover, the drug loading capacity was found to be 6.9% and 6.7% for POE_2_-SH and POE_20_-SH decorated NLCs, respectively.

Other studies of drug-loaded NLCs showed similar drug loading and encapsulation of drugs. For instance, How et al. showed 5.8% drug-loading and 98% drug encapsulation for tamoxifen-loaded NLCs [41]. Similarly, in another study, Faiz et al. confirmed 5.8% drug loading and 83% drug for pioglitazone-loaded nanostructured lipid carriers [19].

### Drug release studies

To evaluate the suitability of the NLCs formulations for sustained drug delivery and the drug release pattern of mucoadhesive APT-loaded NLCs decorated with POE_2_-SH and POE_20_-SH surfactants, the dialysis membrane was used, and PBS was utilized as a dissolution medium maintained at pH 7.4 for 24 h. The dialysis bag method for in-vitro drug release testing is influenced by several limitations. For instance, in some cases, real-time drug release data is not achieved, which can hinder accurate drug burst release [42]. Moreover, the incorporation of surfactants or solvents to enhance the lipophilicity of the dialysis medium may disrupt the structure and stability of the nanocarriers, thus influencing the release profile. However, the dialysis method is extensively utilized due to the ease of sampling, easy media replenishment and mimicking in-vivo conditions [43].

**Fig. 2:**
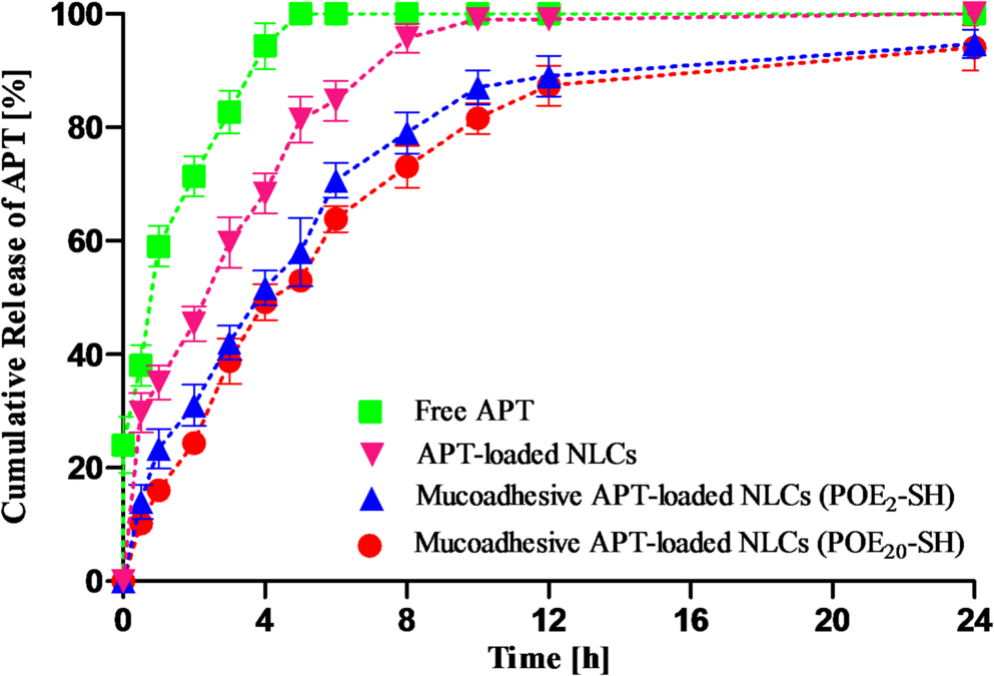
Cumulative percentage release of drug from APT-loaded NLCs, mucoadhesive APT-loaded NLCs decorated with POE_2_-SH and mucoadhesive APT-loaded NLCs decorated with POE_20_-SH via dialysis method

As illustrated in Fig. 2, both short- and long-chain sulfhydryl-modified surfactants containing mucoadhesive APT-loaded NLCs released almost 50% of the drug after 4 h of the study, whereas an initial abrupt drug release was observed from APT suspension. The initial release of the drug can be governed by several factors which include the drug being bonded to the surface, unloaded or submerged in the matrix near to the boundary [19]. With the passage of time, sustained release of the drug was noticed because of gentle matrix hydration. After 5 h of the study, 100% of the free drug was released as compared to both short- and long-chain sulfhydryl-modified surfactants containing mucoadhesive APT-loaded NLCs that provide sustained drug release for 24 h. Moreover, drug release from APT-loaded NLCs was faster than APT-loaded mucoadhesive NLCs.

The encapsulation of lipid nanoparticles contributes to achieve prolonged drug release. There are several parameters, such as drug’s solubility in the lipid, size of particles, the partition coefficient and the type and lipid concentration that could influence the release pattern from NLCs. A sustained drug release is achieved with a rational drug concentration, according to drug release profile [44]. Therefore, because of controlled drug release patterns, NLCs exhibited enhanced permeation.

### Stability studies

For the stability of the formulations, size of particles and entrapment efficiency are two crucial parameters to monitor. After a pre-defined time frame, at 4 ºC and 25 ºC, these parameters were investigated. For POE_2_-SH and POE_20_-SH decorated NLCs, the particle size and PDI were found between 170 and 195 nm and 0.189–0.191, respectively. Furthermore, the entrapment efficiency of 83% for POE_2_-SH containing NLCs and 81% for POE_20_-SH containing NLCs was observed. These findings demonstrate that the long-term storage of the formulation did not cause significant alteration in the compositions of these lipid-based nano-formulations. Moreover, drug-loaded mucoadhesive NLCs containing short- and long chain thiolated surfactants showed almost the same size and PDI after specific time intervals that confirmed the stability of these formulations in simulated gastric and intestinal fluids.

### Safety studies

**Fig. 3:**
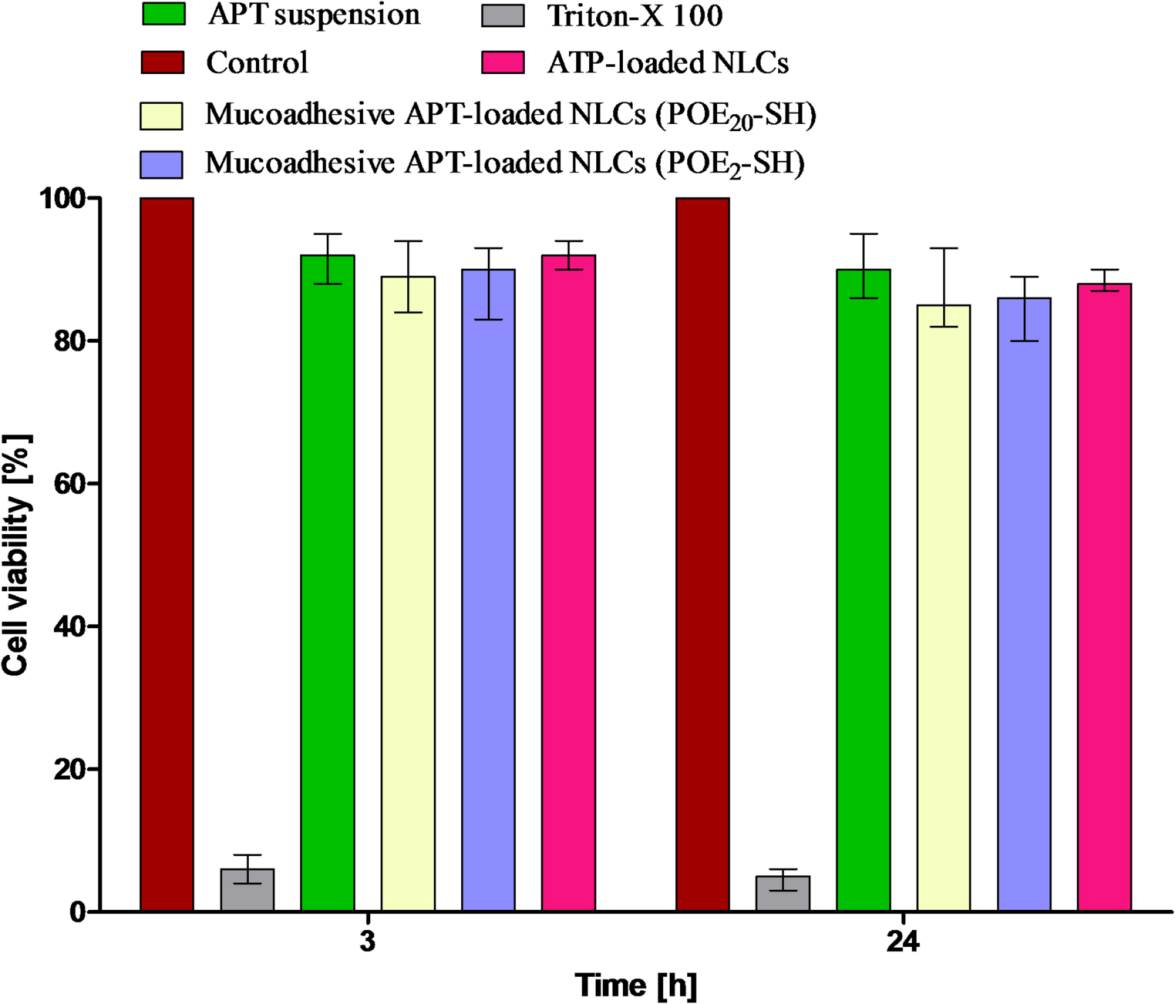
Cell viability (%) of drug-loaded NLCs, mucoadhesive drug-loaded NLCs (POE_2_-SH/POE_20_-SH) in comparison to free drug for a duration of 3 and 24 h at 37 ºC

To examine the effect of mucoadhesive APT-loaded NLCs (POE_2_-SH/POE_20_-SH) on Caco-2 cells, a resazurin assay was conducted. The basic principle of resazurin test depends on the shifting of the dye color in metabolically active cells from oxidized form (blue color) to reduced form (pink color), as the dead cells are unable to reduce resazurin. Therefore, dye acts as an indicator to judge the cell’s viability or toxicity. The mucoadhesive APT-loaded NLCs (POE_2_-SH/POE_20_-SH) displayed > 80% cell viability at 3 and 24 h in comparison to the negative test control, as demonstrated in Fig. 3.

Hence, any indication of significant toxicity was not observed in the newly synthesized formulations. Both sulfhydryl-modified surfactants exhibited safe results for being utilized in NLCs development. The results ensure that the formulations are non-toxic and biocompatible for safe pharmaceutical applications.

### Mucoadhesion studies

The study aims to evaluate the ability of the NLCs formulations to adhere to mucus, enhancing drug retention and bioavailability. The half-cut falcon tube method was conducted using goat intestinal mucosa for investigating the mucoadhesive capabilities of mucoadhesive APT-loaded NLCs comprising POE_2_-SH and POE_20_-SH surfactants. Existence of–SH moieties on the surface of NLCs exhibited mucoadhesive behavior due to the formation of disulfide with the mucus layer [45].

**Fig. 4:**
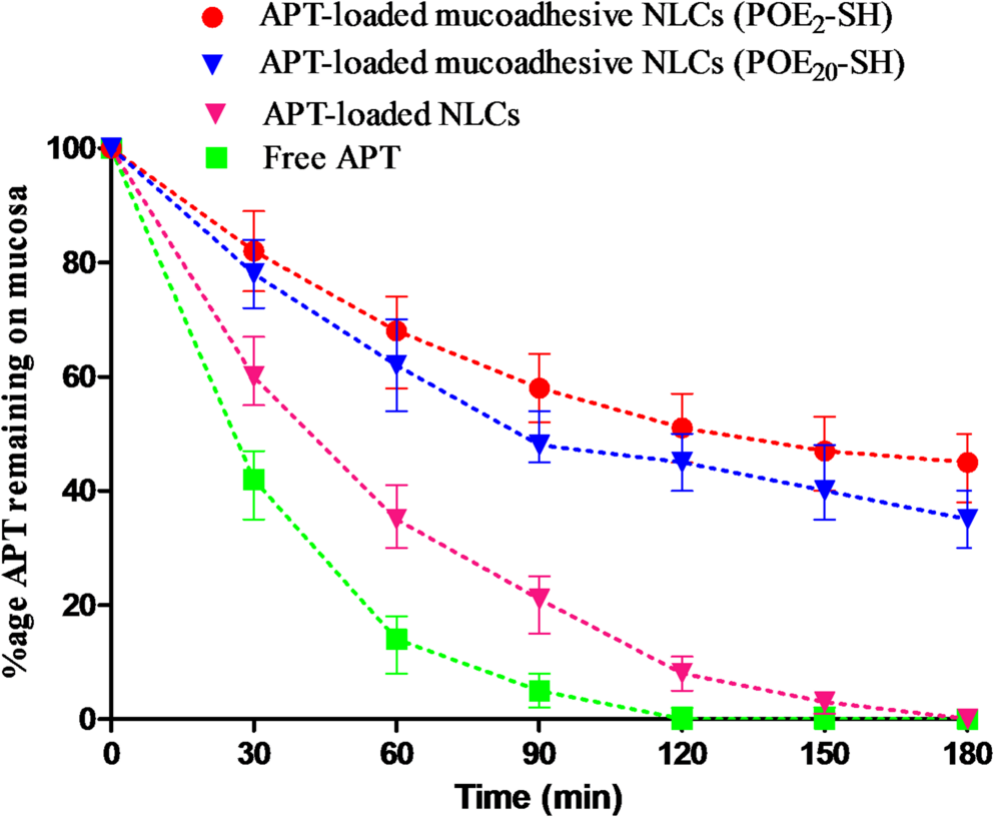
The drug remained on the goat mucosal surface incubated at 37 ºC for free drug, APT-loaded NLCs, mucoadhesive APT-loaded NLCs containing POE_2_-SH surfactant and mucoadhesive APT-loaded NLCs containing POE_20_-SH surfactant

As compared to free drug and drug-loaded NLCs with unmodified surfactants, the mucoadhesive APT-loaded NLCs decorated with POE_2_-SH and POE_20_-SH exhibited improved mucoadhesion as visualized in Fig. 4.

After 1.5 h, mucoadhesive APT-loaded NLCs containing POE_2_-SH and POE_20_-SH surfactants demonstrated 11.6- and 9.6-fold enhanced mucoadhesion in comparison to the free drug, respectively. This increase in mucoadhesion was due to the–SH groups on the surface of NLCs that contribute disulfide bonding with cysteine-rich mucus layer. Therefore, POE_2_-SH and POE_20_-SH containing NLCs were more firmly attached to the mucus leading to enhanced drug concentration on the mucosal surface. Moreover, enhanced mucoadhesion of NLCs decorated with short-chain surfactant in comparison to long-chain sulfhydryl-modified surfactant was due to improved interaction of short-chain surfactant with mucosal layer that causes mucus chain entanglements [9].

### Rheological measurements

**Fig. 5:**
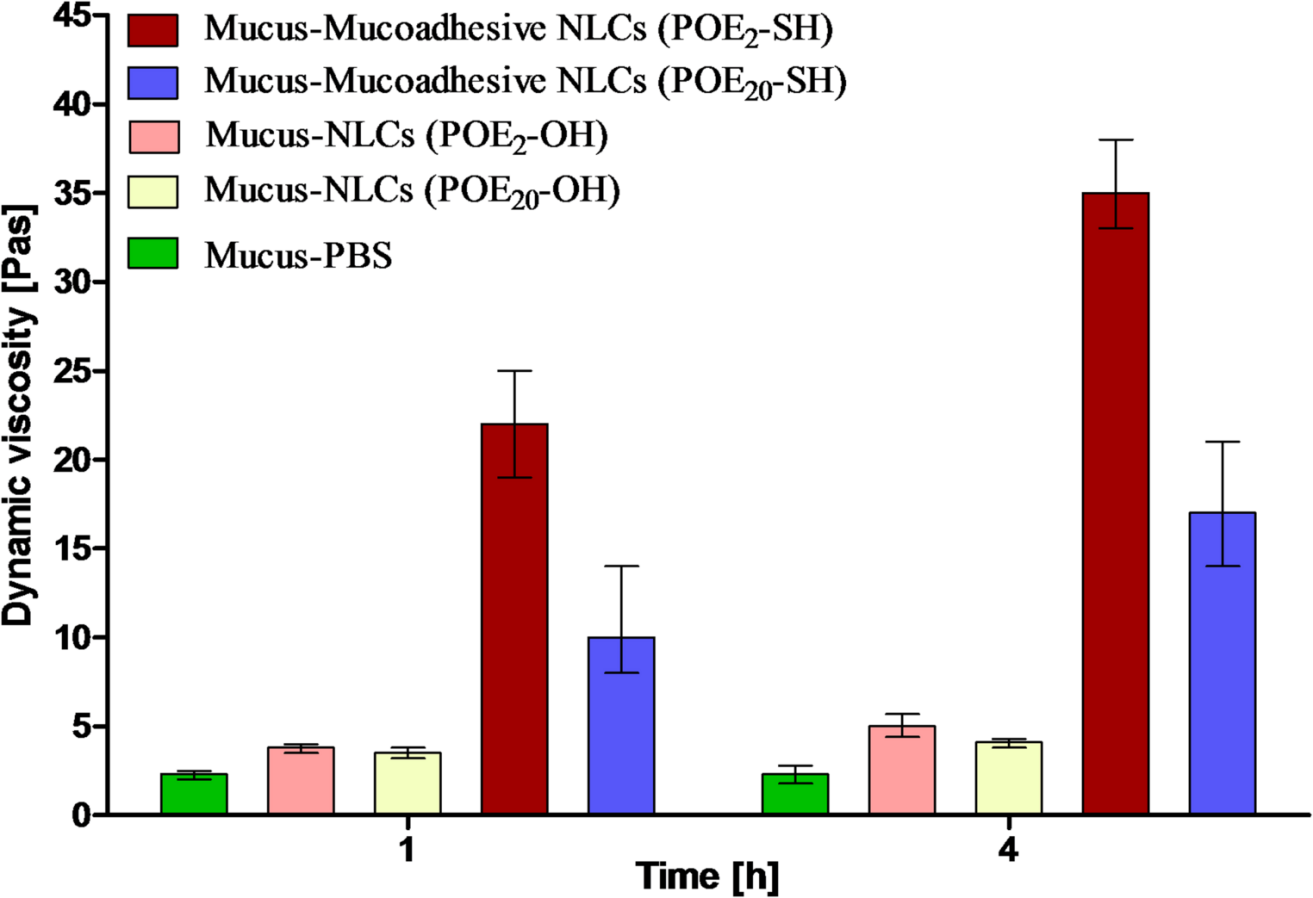
Rheological exploration of different NLCs after incubation at 37 °C with goat intestinal mucus at a frequency of 1 Hz. The given data are means ± SD of at least three experiments

The study aims to assess the interaction between NLCs and mucus, which is crucial for evaluating the strength of mucoadhesion. Rheological studies were performed to evaluate the interaction between NLCs and mucus as illustrated in Fig. 5. Due to the presence of–SH groups on the surface of NLCs, enhanced viscosity was observed.

The mucoadhesive NLCs (POE_2_-SH) showed significantly enhanced viscosity after 4 h, which was 7-fold higher than non-mucoadhesive NLCs (POE_2_-OH), assuming higher interaction of short-chain sulfhydryl-modified surfactants with mucus. This improved interaction between the sulfhydryl-modified surfactants and mucus is usually mediated by non-covalent and covalent bonding between the cysteine-rich mucus and nanoparticles. This disulfide bonding usually occurs immediately after mixing, as confirmed in the results shown. This bonding is strong enough to resist high shear stress and lead to sustained high rheological properties [9].

### Permeation studies

#### Mucus permeation studies

**Fig. 6:**
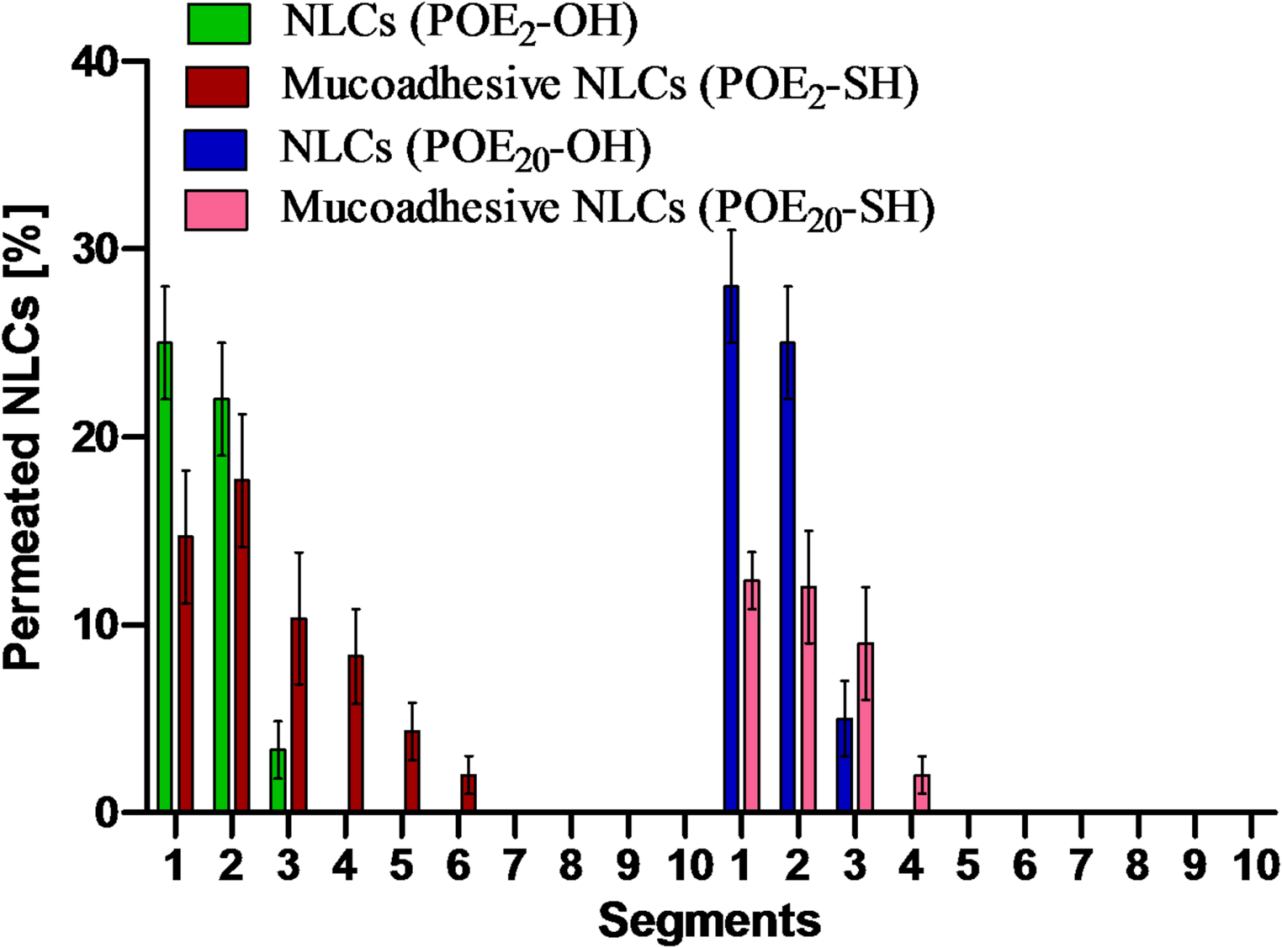
Permeation of fluorescence-labeled different NLCs through goat intestinal mucosa using the rotating tube method

The study aims to evaluate the diffusion potential of mucoadhesive NLCs and how surfactant chain-length affects their ability to penetrate mucus barriers. The ability of short- and long-chain sulfhydryl-modified surfactant-based mucoadhesive NLCs to diffuse through mucus was evaluated via the rotating tube method [28]. The findings of mucus diffusion studies are summarized in Fig. 6. In the first two segments of the silicon tube, the unmodified surfactants containing NLCs showed significantly enhanced permeation as compared to sulfhydryl-modified surfactants containing NLCs. The less diffusion of sulfhydryl-modified surfactants was due to their interaction with mucus via disulfide bond formation. These findings are in agreement with the results obtained from sulfhydryl-modified nanocarriers that exhibited similar behavior [13]. Moreover, in comparison to long-chain sulfhydryl-modified surfactants, short-chain sulfhydryl-modified surfactants are transported into deeper segments of mucus due to less interaction with the mucus. The long-chain surfactant (10 times longer than short-chain) can interfere with mucus more strongly due to long-chain that results in less diffusion.

#### Cell diffusion studies

This study evaluates the ability of sulfhydryl-modified NLCs to enhance drug permeation across cell monolayers by opening tight junctions. The sulfhydryl-modified NLCs improved the diffusion of Lucifer yellow across cell lines in comparison to unmodified surfactants containing NLCs due to the ability of sulfhydryl groups to open tight junctions (TJs) that enhance transport across cell layers. Similarly, in a previous study, sodium fluorescein also showed significantly enhanced via TJs opening by sulfhydryl groups [46].

**Fig. 7:**
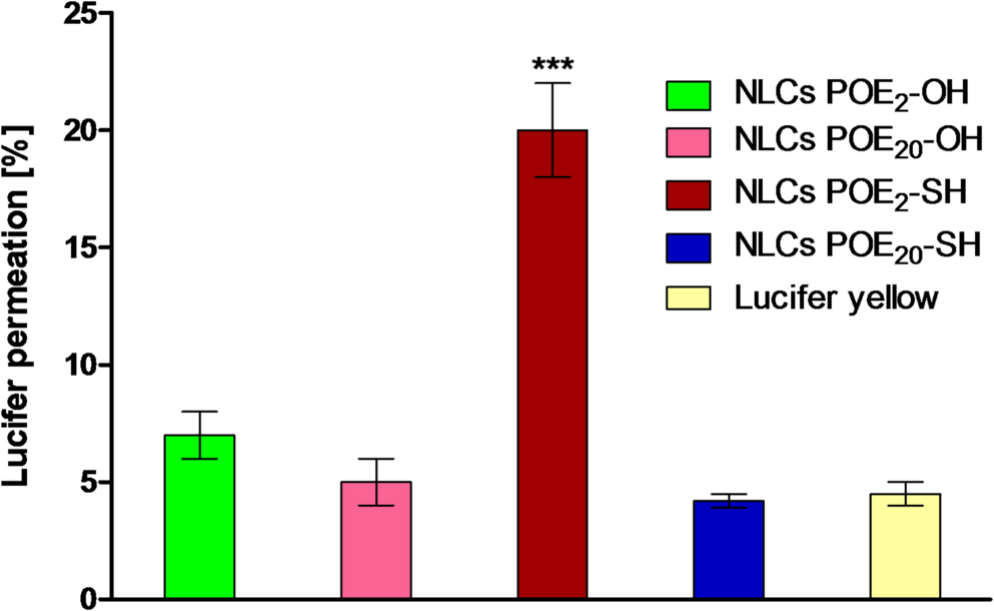
Permeation of fluorescence-labeled NLCs through Coco-2 cell line

The mucoadhesive NLCs decorated with POE_2_-SH exhibited significantly enhanced Lucifer yellow permeation as compared to POE_20_-SH due to increased intensity of short-chain sulfhydryl-modified surfactant to open TJs. Moreover, a smaller number of–SH groups are decorated on the surface of long-chain surfactants due to high molecular weight and this concentration of–SH groups are insufficient to open the significant number of TJs essential for diffusion (Fig. 7).

**Table 3:** Comparison of the permeation effect of fluorescence-labeled short-chain sulfhydryl-modified surfactant with long-chain sulfhydryl-modified surfactants and transport enhancement ratio (R) on Caco-2 cell monolayer (mean ± SD, *n* = 3)

Sample	Apparent permeability coefficient	Transport enhancement ratio
	[*P*_app_ × 10^− 6^ (cm/sec)]	*R*= [*P*_app_(sample)/*P*_app_(control)]
Lucifer yellow	2.25 ± 0.3	1
NLCs POE_2_-OH	2.50 ± 0.4	1.11
NLCs POE_20_-OH	2.28 ± 0.6	1.01
NLCs POE_2_-SH	5.29 ± 0.5	2.35
NLCs POE_20_-SH	3.29 ± 0.8	1.46

### Proof-of-concept studies

**Fig. 8:**
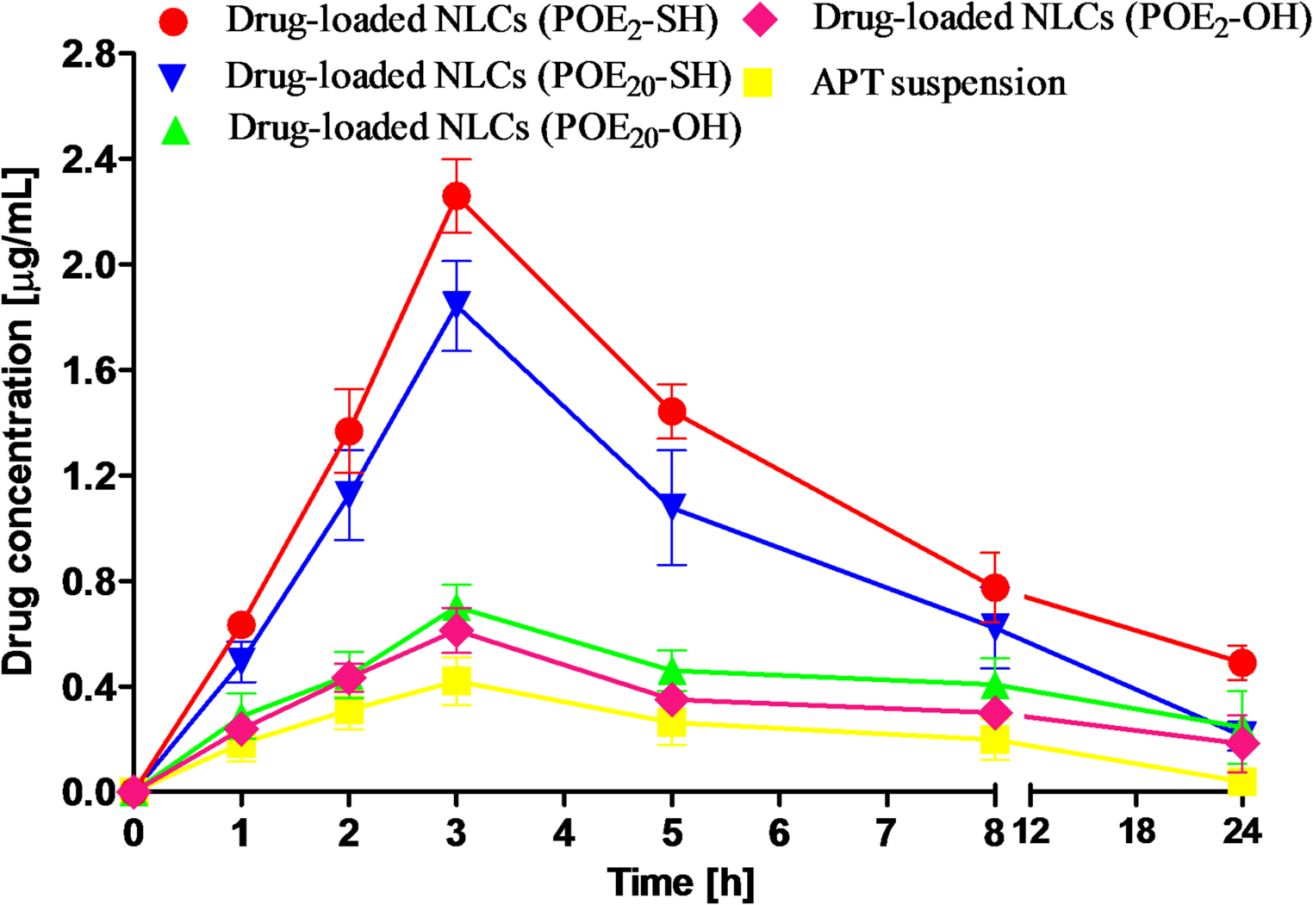
The drug profile of plasma concentration vs. time with an equal amount of drug in a suspension, drug-loaded NLCs with unmodified and short- and long-chain sulfhydryl-modified surfactants that were orally administered to four groups of SD rats (*n* = 5). The fifth group (*n* = 3) received the same drug concentration intravenously

The study aims to evaluate the pharmacokinetic performance of NLCs decorated with short- and long-chain sulfhydryl-modified surfactants to enhance oral bioavailability of aprepitant. The plasma drug concentration versus time data of drug suspension, drug-loaded NLCs decorated with short- and long-chain sulfhydryl-modified surfactants are shown in Fig. 8. Moreover, the other pharmacokinetic parameters are revealed in Table 4.

**Table 4:** PK parameters of drug suspension, drug-loaded unmodified surfactant containing NLCs and short- and long-chain sulfhydryl-modified surfactants containing NLCs obtained after non-compartmental analysis by PKSolver

Parameters	Formulations				
	Drug	NLCs (POE_2_-OH)	NLCs (POE_20_-OH)	NLCs (POE_20_-SH)	NLCs (POE_2_-SH)
Dose (mg/Kg)	2.0	2.0	2.0	2.0	2.0
T_max_ (h)	2.91 ± 0.7	3.0 ± 0.7	3.1 ± 0.7	3.0 ± 0.5	3.0 ± 0.4
C_max_ (µg/mL)	0.42 ± 0.03	0.61 ± 0.02	0.70 ± 0.02	1.84 ± 0.04	2.26 ± 0.08
AUC_0 − 24_ (µg/mL*h)	8.11 ± 0.6	10.9 ± 0.4	11.1 ± 0.5	20.85 ± 0.9	29.2 ± 1.2
^a^Fr (%)	9.67	13.0	13.2	24.87	34.8

^a^ AUC after 2.0 mg intravenous administration of drug was 83.84 ± 08 µg/mL* h

The drug-loaded NLCs decorated with short- and long-chain sulfhydryl-modified surfactants demonstrated significant enhanced C_max_, and AUC leading to improved drug bioavailability in comparison to free drug suspension and unmodified surfactants containing NLCs. Moreover, due to better permeation properties of short-chain sulfhydryl-modified surfactants containing NLCs, better bioavailability was shown by these surfactants.

APT is a BCS Class IV drug, so after oral administration, poor plasma drug concentration was observed after 3 h (C_max_ 0.42 µg/mL) representing poor drug bioavailability. On the contrary, short- and long-chain sulfhydryl-modified surfactants decorated NLCs exhibited significantly enhanced AUC and C_max_ after 3 h of oral administration. However, the long-chain sulfhydryl-modified surfactants showed 4.38-fold enhanced C_max_, whereas due to better diffusion properties, the short-chain sulfhydryl-modified surfactants exhibited 5.38-fold enhanced C_max_. Similarly, 34.8% of relative bioavailability (F_r_) was attained for short-chain surfactants and 24.8% for long-chain surfactants. A similar bioavailability enhancement was observed in another study of aprepitant-loaded solid dispersion [47]. In contrast to previous findings, short-chain sulfhydryl-modified surfactants were used first the time to enhance aprepitant oral bioavailability.

The significant improvement in oral APT bioavailability of these short-chain modified surfactants is due to several factors. Firstly, the sulfhydryl-modified surfactants enhance mucoadhesion properties, leading to enhanced gastric residence time as evident in enhanced C_max_ and AUC that contribute to enhanced drug diffusion [24]. Secondly, sulfhydryl-modified surfactants enhance the drug diffusion due to the opening of TJs, as evidenced by in-vivo findings. Thirdly, short-chain sulfhydryl-modified surfactants have a more significant effect on permeation than long-chain and more improved oral drug bioavailability was observed.

## Conclusion

Recently, nanostructured lipid carriers (NLCs) decorated with sulfhydryl-modified surfactants have attracted significant attention for being used for the delivery of BCS Class IV drugs. However, the impact of the chain-length of these surfactants on different properties of NLCs is still unknown. Therefore, in this study, a comparative analysis was made between short- and long-chain sulfhydryl-modified surfactants decorated with mucoadhesive NLCs. For this purpose, short- and long-chain sulfhydryl-modified polyethoxylated surfactants were generated to develop mucoadhesive NLCs and loaded with APT. The long-chain sulfhydryl-modified surfactants showed 4.38-fold enhanced C_max_, whereas due to better diffusion and mucoadhesion properties, the short-chain sulfhydryl-modified surfactants exhibited 5.38-fold enhanced C_max_. Similarly, 34.8% relative bioavailability was attained for short-chain surfactants and 24.8% for long-chain surfactants. Based on these findings, it can be concluded that short-chain sulfhydryl-modified surfactants are more suitable nanocarriers for the delivery of poorly soluble drugs.

## Electronic supplementary material

Below is the link to the electronic supplementary material.

Supplementary Material 1

## Data Availability

Data can be provided by the authors upon request.
