# Supplementary material for: Impact of chain-length of sulfhydryl-modified surface-decorated surfactants on mucoadhesive nanostructured lipid carriers
Source: Drug Deliv Transl Res. 2025 Jun 24;16(2):549–62. doi: 10.1007/s13346-025-01905-w (PMC12819570; doi:10.1007/s13346-025-01905-w)
Supplement: Supplementary file 1 — Supplementary Material 1 [file 13346_2025_1905_MOESM1_ESM.docx]

**Supplementary Data**

**Impact of chain-length of sulfhydryl-modified surface-decorated surfactants on mucoadhesive nanostructured lipid carriers**

Samia Kausar^1,2^, Sofia O. D. Duarte^3,4^, Ahmed Raza Hashmi^5^, Farwa Zahra^6^, Alia Erum^1,*^, Shumaila Arshad^7^, Ume Ruqia Tulain^1^, Mulazim Hussain Asim^1,*^ Pedro Fonte^3,4,8,9,*^

^1^College of Pharmacy, University of Sargodha, 40100-Sargodha, Pakistan

^2^Lahore College of Pharmaceutical Sciences, Raiwind road, 54000-Lahore, Pakistan

^3^iBB – Institute for Bioengineering and Biosciences, Department of Bioengineering, Instituto Superior Técnico, Universidade de Lisboa, Av. Rovisco Pais 1, 1049-001 Lisboa, Portugal

^4^Associate Laboratory i4HB—Institute for Health and Bioeconomy at Instituto Superior Técnico, Universidade de Lisboa, Av. Rovisco Pais, 1049-001 Lisboa, Portugal

^5^School of Pharmacy, Monash University Malaysia, Bandar Sunway, Subang Jaya, Selangor Malaysia

^6^Doctorʹs Institute of Health Sciences, 40100-Sargodha, Pakistan

^7^ILM College of Pharmaceutical Sciences & Sargodha College of Medical Sciences, 40100-Sargodha, Pakistan

^8^Department of Chemistry and Pharmacy, Faculty of Sciences and Technology, University of Algarve, Gambelas Campus, 8005-139 Faro, Portugal

^9^Centro de Ciências do Mar do Algarve (CCMAR/CIMAR LA), Campus de Gambelas, Universidade do Algarve, 8005-139 Faro, Portugal

***Corresponding authors:**

Dr. Mulazim Hussain Asim

e-mail: [mulazim.hussain@uos.edu.pk](mailto:mulazim.hussain@uos.edu.pk)

College of Pharmacy, University of Sargodha, 40100-Sargodha, Punjab, Pakistan

Dr. Alia Erum

e-mail: [alia.erum@uos.edu.pk](mailto:alia.erum@uos.edu.pk)

College of Pharmacy, University of Sargodha, 40100-Sargodha, Punjab, Pakistan

Prof Dr. Pedro Fonte

e-mail: [prfonte@ualg.pt](mailto:prfonte@ualg.pt)

Faculty of Sciences and Technology, University of Algarve, Gambelas Campus, 8005-139 Faro, Portugal


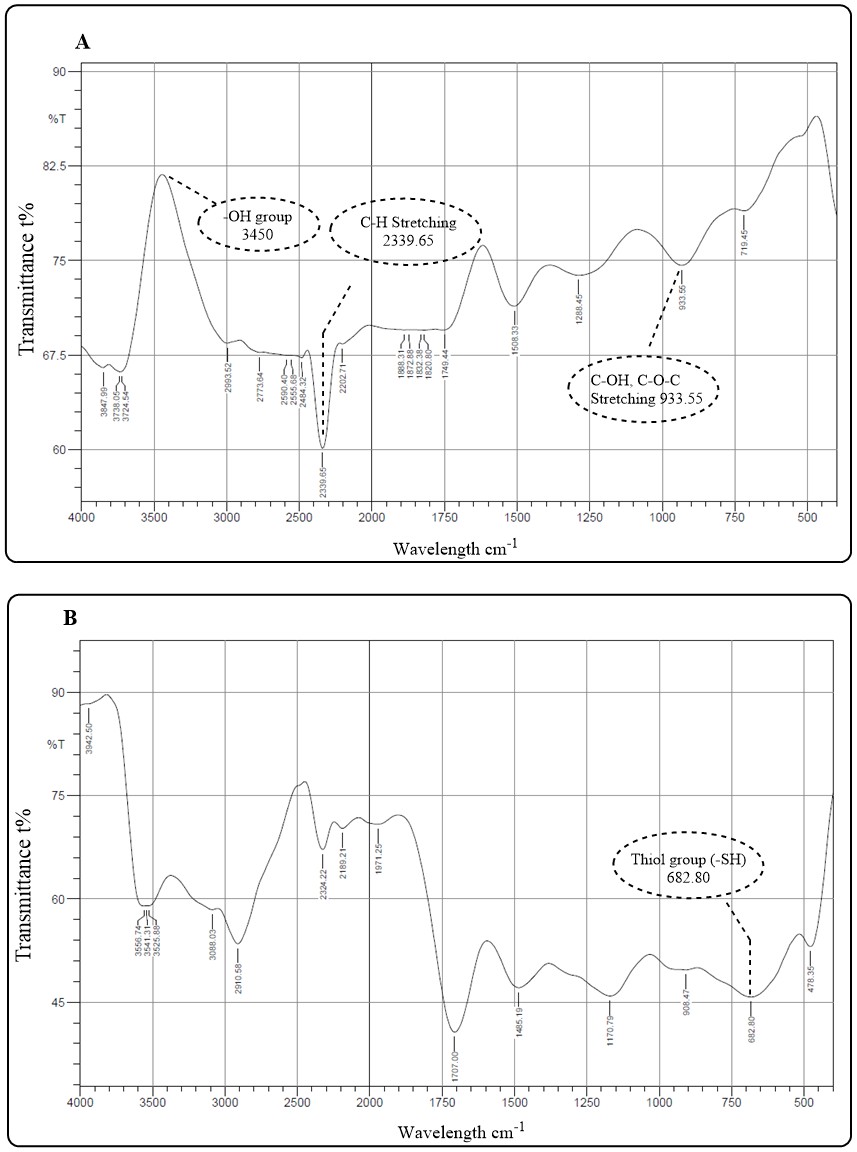
**Supplementary Figure S1:** FT-IR spectra of **[A]** polyoxyethylene (2) oleyl ether (POE_2_-OH) and **[B]** sulfhydryl-modified polyoxyethylene (2) oleyl ether (POE_2_-SH)


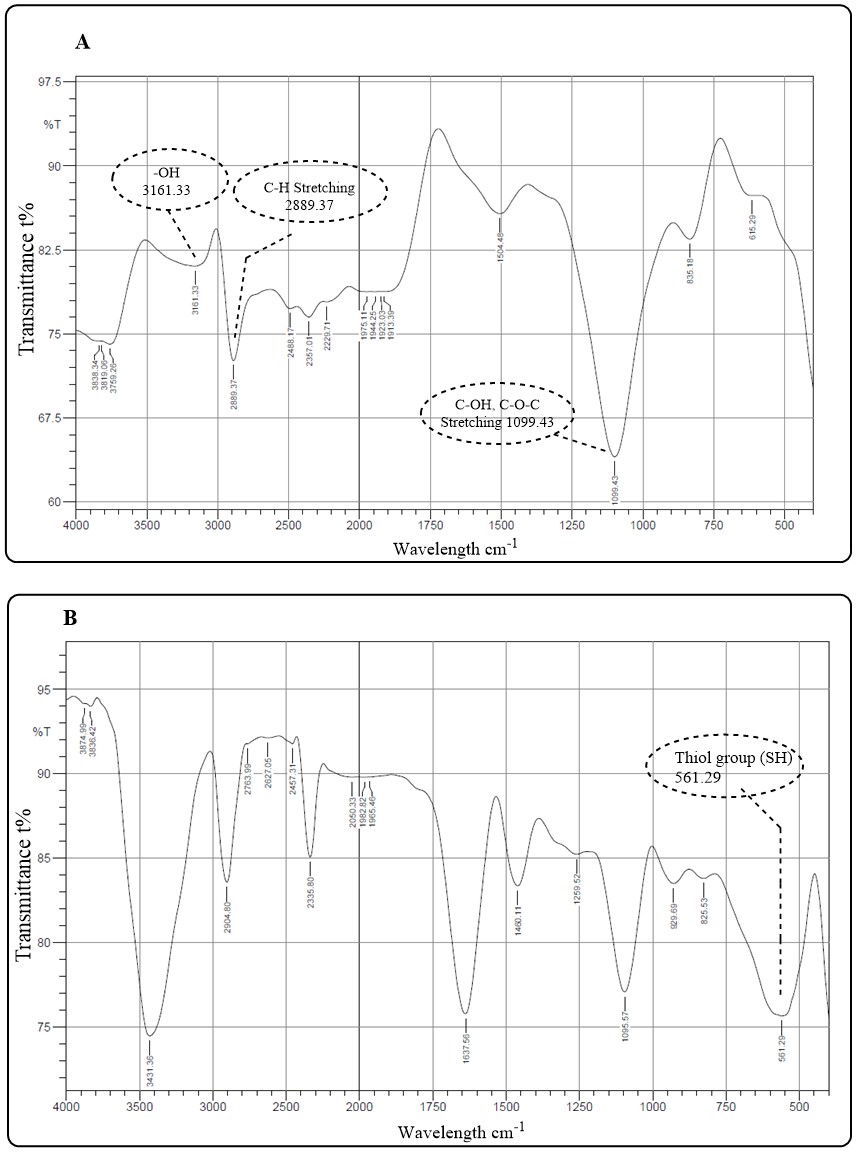


**Supplementary Figure S2:** FT-IR spectra of **[A]** polyoxyethylene (20) oleyl ether (POE_20_-OH) and **[B]** sulfhydryl-modified polyoxyethylene (20) oleyl ether (POE_20_-SH)


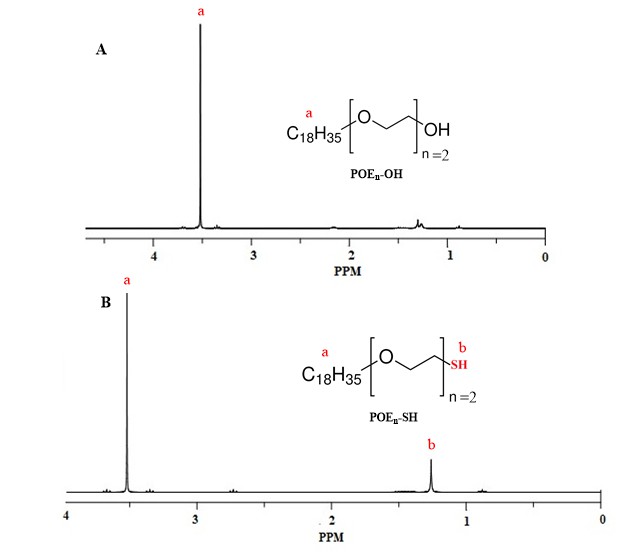


**Supplementary Figure S3: ^1^**H NMR of **[A]** polyoxyethylene (2) oleyl ether (POE_2_-OH) and **[B]** sulfhydryl-modified polyoxyethylene (2) oleyl ether (POE_2_-SH)


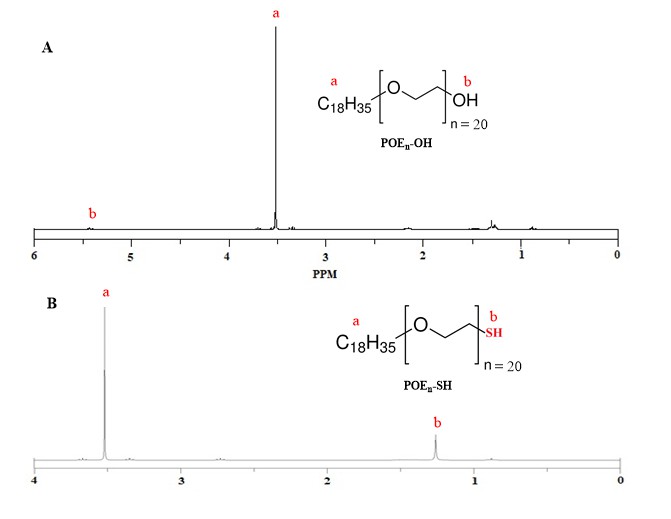


**Supplementary Figure S4: ^1^**H NMR of **[A]** polyoxyethylene (20) oleyl ether (POE_20_-OH) and **[B]** sulfhydryl-modified polyoxyethylene (20) oleyl ether (POE_20_-SH)
